# Weather extremes and their impact on crop transportation networks: Evidence from U.S. Midwestern elevators

**DOI:** 10.1371/journal.pone.0319815

**Published:** 2025-03-31

**Authors:** Theodoros Skevas, Wyatt Thompson, Benjamin Brown, Delmy Salin, Jesse Gastelle, Edgar Marcillo-Yepez

**Affiliations:** 1 Division of Applied Social Sciences, University of Missouri, Columbia, Missouri, United States of America; 2 Transportation Economics Division, U.S. Dept. of Agriculture, Transportation and Marketing Program, Agricultural Marketing Service, Washington DC, United States of America; University of Vienna: Universitat Wien, AUSTRIA

## Abstract

The grain price margins between buyers and sellers (i.e., basis spread) is influenced by the infrastructure used to transport crops from collection points to ports, which can be disrupted by weather extremes like floods and severe storms. Such disruptions are expected to become more frequent, potentially increasing food insecurity and impacting farm incomes. On average, the U.S. accounts for one-third of global corn and soybean production from 2012/13 to 2020/21, so the infrastructure to move crops from the main growing region to the nation’s ports is critical to global crop and food markets. Despite the critical nature of these issues, there is limited research specifically examining the effects of weather extremes on the U.S. crop transportation network. This study investigates how weather extremes disrupt crop transportation networks, and, in turn, how those disruptions affect the basis spread of corn and soybeans. It uses basis spread data from nearly 5,000 U.S. midwestern corn and soybean elevators spanning from 2012 to 2020, along with natural disaster declarations to represent weather extremes affecting crop transportation. Using a three-step process, it calculates least cost transportation routes to a port, adjusts for weather disruptions, and integrates disaster, transportation cost, and control variables into a fixed effects, panel data model that explains variation in basis spread. Results show natural disasters, particularly flash floods and winter storms, negatively affect basis spread. The cost effects of natural disasters disrupting crop transportation routes further decrease basis spread. Strengthening crop transportation infrastructure to withstand flooding and winter storms could reduce disruptions in this network. These findings underscore the value of Federal and State policies that prioritize investments in resilient transportation infrastructure, particularly in regions prone to flash floods and winter storms. Strengthening this infrastructure could not only reduce the economic costs of weather disruptions but also affect farm income and food security.

## 1. Introduction

The distribution network connecting crop production to final users plays a key role in determining global food security and rural economic development, and can be assessed in terms of export competition, consumer price implications, and producer return effects [1]. This network can be disrupted by weather extremes, such as floods that inundate railroads and winter storms that ice over roads. While the weather extremes are known to imperil crop distribution systems, we are aware of no previous study that quantifies these impacts as we do here. We estimate how weather extremes affect the crop distribution system to assess how climatic conditions affect the costs of moving crops from producers to consumers, with immediate implications for consumer food costs and producer returns and broader importance for food security and economic development.

Our experiments focus on the connection between a primary growing region and a key market. The Midwest region of the U.S., often called the Corn Belt, with states such as Illinois, Iowa, Minnesota, Nebraska, Ohio, Indiana, and Missouri is one of the most important growing regions [2]. The U.S. accounts for an average of 33% of world corn production and 32% of world soybean production in 2012/13-2020/21 [3]. The distribution networks for these crops rely on an integrated multimodal transportation system, including a combination of highways, railroads, and waterways [4]. Key ports serve as vital hubs and the Mississippi River and its tributaries are critical for transporting large volumes of corn and soybeans to Gulf Coast ports for export [5]. A shock to this system that raises the costs of moving crops can be passed on to buyers in the form of a higher price they pay or to inland producers and handlers sellers in the form of a lower selling price.

Basis spread between the prices at collection points in the interior of the United States and the port depends in part on the infrastructure of moving grain from these locations to destinations. Prices at both end-points are important: the price at Midwest collection points are the prices that producers get for selling their crops, so this is the supply inducing price; and the export market price is a key indicator of overall price conditions for processors worldwide, foreshadowing the consumer prices for finished goods. Scientists have long studied crop basis, which can include both basis spread and intertemporal impacts, including by conducting empirical analysis to detect the impacts of fuel costs, local supplies and demands, export volumes, and other factors that typically omit weather variables [6–11]. One study estimates the impacts of persistent temperature and precipitation patterns on crop basis spread, finding that variables that reflect weather at weekly frequency affect basis spread by affecting the costs of river, rail, and road travel [12]. We are aware of no quantitative estimates of the impact of weather extremes on transportation costs. This potential that the infrastructure that supports this movement of grain is vulnerable to disruption by weather extremes and natural hazards, such as floods that can submerge bridges and railroads, and severe winds, hail, or landslides that can slow or halt traffic has been recognized [5]. Moreover, scientists find that such weather extremes could become more frequent in the future [13,14], leading to greater costs of planning, network operations, maintenance, and vehicle performance, with more canceled or rerouted trips [14–16]. These disruptions would also affect the crop transportation network. However, the lack of scientific study means that decision makers who consider investments in key aspects of the infrastructure system, such as road bridges and river locks, might be unable to quantify the value of steps that reduce these risks.

Empirical analysis can estimate how weather extremes affect transportation costs in terms of the dollars per bushel shipped. Our method is consistent with the state of the art of scientific study on basis spread [7–9,11–12]. We adapt methods of this literature to the problem at hand, with recently published research quantifying the impacts of weekly precipitation and temperature impacts on basis spread a key reference. Basis spread studies do not take weather extremes into account; most existing studies investigated the impact of weather extremes on farm production and prices [17–25], not the transportation network, so this literature is only loosely related. Some research has examined the impact of weather extremes and climate change more generally on the transportation infrastructure in the U.S [15,16,26,27]. and in non-U.S. contexts [28–30]. These studies did not address the specific influence on crop distribution networks so they do not help with key questions such as how to handle the unique and widely distributed collection sites throughout the region, the specific problems associated with available datatypes, or seasonal factors. One study examined the effects of climate change on U.S. grain transport, focusing on broader aspects like crop production shifts, Great Lakes water levels, and navigation possibilities [31], but without quantifying the direct impact of weather extremes on crop distribution network costs or spreads. The fact that these studies generally conclude that extreme weather events are likely to adversely affect the transportation infrastructure usefully confirms that our findings fit into the broader literature, yet give few insights about data or problem specification.

This article generates novel estimates of how weather extremes affect the U.S. crop distribution system. We map the stylized least-cost routes of the U.S. crop distribution network, identify instances when extreme weather interferes with that mapping and find an alternative route in these cases, and estimate the impacts of these disruptions on the crop basis spread in terms of the dollars per bushel cost increases. We find that weather extremes have caused greater costs to the crop distribution network in the sample period, as shown below. This finding coupled with other scientists’ findings that weather extremes will become more frequent highlight the relevance of this research. Weather extremes have real effects on basis spread, meaning the gap between buyer and seller prices widens. These consequences harm crop producers and sellers, potentially leading to lower farm income and reduced economic activity in rural areas on the one hand and higher food costs and lower food security on the other hand – although these broader implications go beyond the scope of this article. Quantifying the threats of extreme weather to the crop distribution infrastructure provides information relevant to decision makers who consider future investments in this system. The value of investments to reduce ice on a road bridge, protect a railroad in low area from flash floods, or increase the tolerance of a river lock for water surges during a flood might depend in part on the costs caused by weather extremes.

The remainder of this study is organized as follows: section 2 describes the methods used to conduct the analysis. Data are discussed in section 3. Estimation results are detailed in section 4, followed by conclusions and discussion in section 5.

## 2. Overview of the methods

To assess the effect of weather extremes on crop basis spread, a three-step procedure was used. First, hypothetical least cost-routes were developed to move crops from sample elevators to the port of New Orleans, Louisiana (NOLA). In the second step, these routes were re-calculated to account for disruptions caused by natural disasters blocking transportation pathways. Finally, using the adjusted transportation routes and natural disaster indicators from step two, an econometric model was estimated, incorporating control variables, to quantify the impact of weather extremes on crop basis spread. Each of these steps is described in more detail below.

### 2.1. Developing hypothetical least-cost routes for transporting crops

**Fig 1:**
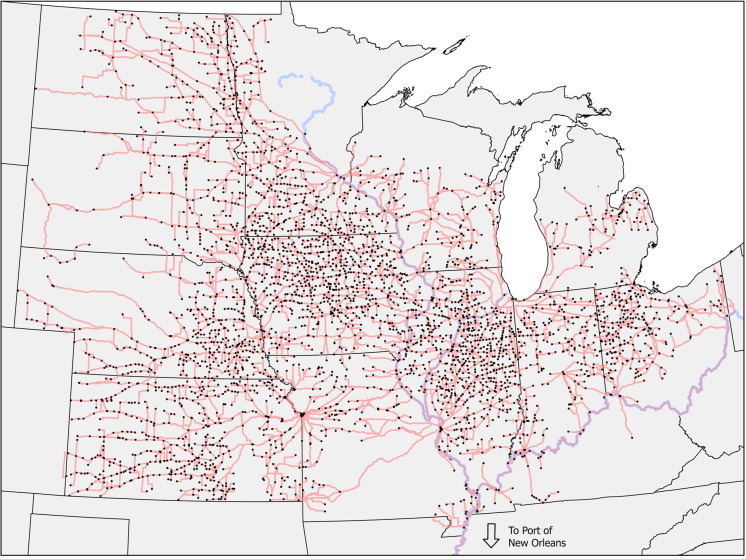
Least-cost routes of sample elevators (without disaster constraints). Elevators are depicted as circles. Red lines indicate the least-cost transportation routes for each elevator, utilizing either road or rail infrastructure. Blue lines represent rivers within the transportation network. The hierarchy of transportation modes prioritizes rivers over railways and railways over roads, reflecting their relative cost-effectiveness for grain transport.

Least-cost routes for transporting crops to the port of New Orleans were computed using Geographic Information System (GIS) techniques. The first step in this process was to combine the coordinates of sample elevators with geospatial data on roads, railways, and navigable rivers (i.e., Mississippi, Ohio, and Illinois Rivers). These combined data were then used to create a network dataset with roads, railways, and rivers as edges, and grain elevators as nodes. It is common for a bushel of grain to use at least two of these transportation modes before reaching an end user [32]. The following cost levels were associated with each type of edge: 0.1/mile for truck, 0.001/mile for rail, and 0.00001/mile for barge. These assumed costs were used to enforce the following hierarchy of transportation modes: 1) rivers, 2) railways, and 3) roads. This hierarchy puts crops on rail instead of road, and on river instead of rail to reflect relative transportation cost as one moves along the hierarchy. This hierarchy reflects the fact that barge is the most cost-effective mode of grain transportation from the upper Mississippi River basin to NOLA [33]. At least two-thirds of U.S. corn and soybean exports are transported by barge, with inland waterway transport accounting for about half of the U.S. grain exports [32,34]. The base (i.e., disruption free) least cost routes of sample elevators is shown in **Fig 1**. The assumed costs used to determine the hierarchy of transportation modes is not used to calculate the implied transportation cost for each route. Instead, weekly national average costs of shipping crops are estimated from available historical data. The product of these values and usage of each mode drive the transportation cost variable.

It is important to note here that the generated routes are simulated least-cost routes for goods leaving the sample elevators for New Orleans exclusively via the Mississippi River. We acknowledge that the actual routes can differ because (a) costs are not common among all routes of a type, with each span of road, rail, or river probably having at least some difference in cost, (b) not all crops go via the Mississippi River to New Orleans, with any amount moving out of the region in a different direction or even used in the region, although that’s not directly relevant to our look into the price spreads relative to the port, and (c) there will always be some error.

### 2.2. Hypothetical least-cost solutions accounting for natural disasters

**Fig 2:**
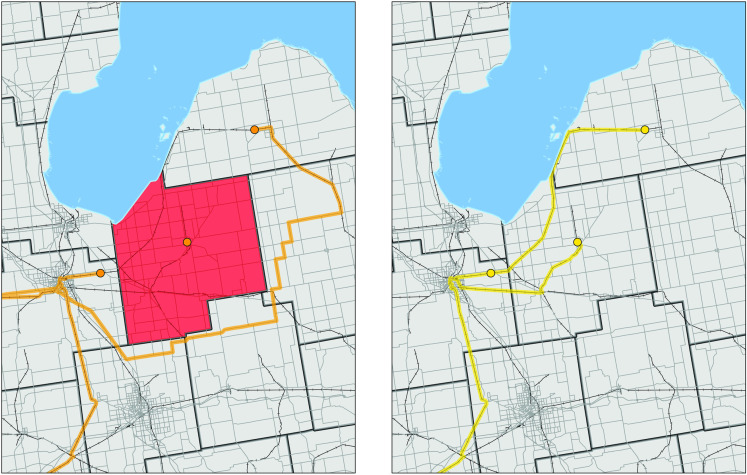
Example of a natural disaster occurring at the elevator’s county of operation. Elevators are shown with circles. Yellow and gold lines show least-cost routes for scenarios with (left panel) and without (right panel) disruptions. The county in red acts as a barrier due a natural disaster occurring in that county in a particular week.

To identify disruptions blocking the crop transportation pathways, the elevator-specific least-cost routes developed in the previous step were overlaid onto maps of county-specific weekly natural disaster data (see the Data section for details on natural disasters). Natural disasters can occur at the county where an elevator is located and/or along its least-cost route (developed in step 1). In the first case, no route is drawn to the elevator the week that the disaster took place, consistent with the possibility that disasters at the collection point tend to disrupt transactions. For disasters occurring along the route, a new route bypassing the affected counties is drawn. This procedure is depicted in **Fig 2**. The left image in this figure shows the least-cost (undisrupted) routes of three elevators (depicted by circles). The right image shows how the least-cost routes of the same three elevators are affected when the county in red acts as a barrier due a natural disaster occurring in that county in a particular week. No route is drawn to the elevator in the center of the affected county. For the elevator located to the northeast of the affected county, a new route (heading south, and then west) bypassing the affected county is drawn. For the elevator located west of the affected county, its route is not impacted by the natural disaster. Once again, weekly national average transportation costs by mode are combined with the transportation path after the disruption to estimate the implied cost effects. The least-cost solution with disaster constraints allows the calculation of elevator-specific crop transportation costs that account for the impact of natural disasters. It further allows the identification of the type of disaster affecting crop transportation and whether the disaster occurred at the elevator point or along the route.

### 2.3. Basis spread regression

The effects of weather extremes on crop basis spread of grain collection points in the central U.S. states were assessed using an econometric panel data model. This model explains changes in basis spread as a function of crop transportation cost, weather extremes (i.e., natural disasters), and control variables. Estimation of basis spread is performed using the equation below:


$$B_{i,tw}=\beta_{0}+\beta_{1}C_{i,tw}+\beta_{2}D_{i,tw}+\beta_{3}X_{i,tw}+\gamma_{i}+\epsilon_{i,tw}$$ (1)

where $B_{i,tw}$ is the weekly average commodity basis spread quoted by elevator *i* in week *w* of year *t*, $C_{i,tw}$ is the shipping cost along the hypothetical least-cost route that accounts for natural disasters, $D_{i,tw}$ are disaster dummies indicating whether a natural disaster occurred at the elevator’s county of operation, $X_{i,tw}$ is a vector of control variables, $\varepsilon_{i,tw}$ is an error term, the betas are parameters to be estimated, and $\gamma_{i}$ captures time-invariant unobserved differences across elevators. To determine whether $\gamma_{i}$ are treated as fixed or random effects a Hausman test was employed. This test examines whether the individual effects $\gamma_{i}$ are correlated with the independent variables in the model. The null hypothesis assumes no correlation, favoring the random effects model, while the alternative suggests correlation, favoring fixed effects. The test follows a Chi-square distribution with *k* degrees of freedom, where *k* is the number of independent variables. If the test statistic exceeds the critical value, we reject the null and select the fixed effects model; otherwise, the random effects model is preferred. A model that ignored unobserved heterogeneity $\gamma_{i}$ was also estimated for comparison purposes. Control variables include crop production and ethanol intensity (described in more detail in the Data section). Control variables are selected based on findings in the literature about other factors that can drive crop distribution costs, such as local demand and supply conditions, adapted to the present context of weather extremes and basis spread that already includes location-specific transportation price indicators [7–12]. Equation (1) was estimated separately for corn and soybeans. We include an ‘aggregate’ disaster dummy in the basis spread estimation as an initial test for evidence of the impact of any type of natural disaster on basis spread. We also re-estimate the basis spread model with separate natural disaster dummies to assess the effect of individual disasters on basis spread. Model 1 is also re-estimated by including the interaction term of the recalculated shipping cost and a dummy variable indicating whether the route has changed because of a disaster hitting the route. The route change dummy was not included in estimations because it was found to be almost perfectly correlated with its interaction with the recalculated shipping cost variable. This interaction variable could help see if disruptions are less or more serious than implied by the shipping cost calculations. For example, a negative value could measure if shippers can mitigate the cost impact implied by the rerouting through other responses, such as storage or selling to a different end user on a different path.

The cost of moving a crop to market is expected to have a negative impact on basis spread. Higher shipping costs lead to lower local price for a given port price. A disaster at the collection point would tend to disrupt transactions, but the effect on basis spread is uncertain. If the marginal flow is from crop stored at the collection point out to broader markets, then inability to move crops out might push down basis. If the marginal flow is from fields or neighboring collection points to the collection point in question, then the inability to move crops in might push up basis. Crop production is an indicator of local supply and demand conditions around the collection point. Higher local supply suggests more supply pushes crops through the system and is expected to decrease basis. Finally, ethanol production around the collection point should raise basis (i.e., increase local price relative to a given port price) for corn and likely for soybeans, too. More local use of crop means less stress on the system to move the crop to the port for export.

## 3. Data

**Fig 3:**
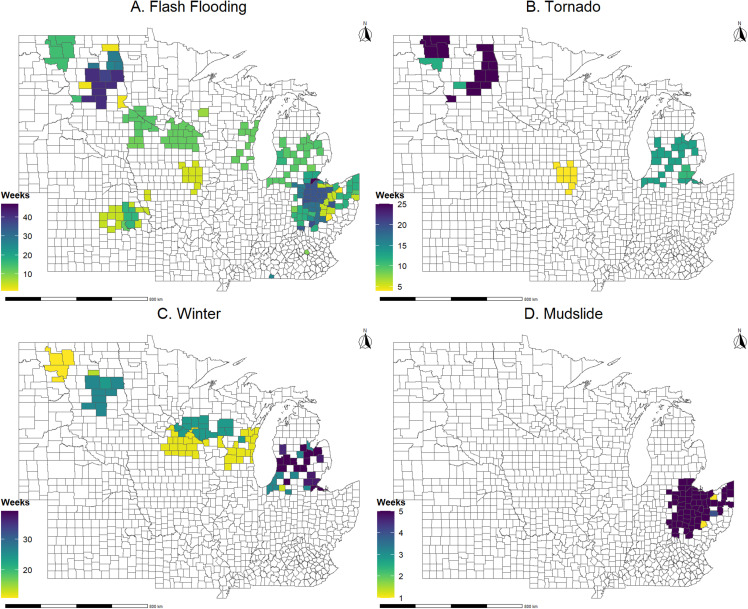
Mapping the frequency of flash floods, mudslides, tornadoes, and winter storms, 2012 – 2020.

Data on corn and soybean basis spread, grain collection points’ location, natural disasters, data on transportation costs of corn and soybeans, and data on control variables over the period 2012-2020 were collected for all states of the upper Mississippi River basin. These states are Iowa, Illinois, Indiana, Kansas, Kentucky, Michigan, Minnesota, Missouri, North Dakota, Nebraska, Ohio, South Dakota, and Wisconsin. The basis spread data are daily observations (transformed to weekly values) for 2,593 and 2,304 corn and soybean elevators and were retrieved from Refinitiv Thomson Reuters database. The geographic distribution of sample elevators is shown in **Fig 1**. Natural disaster data are county-specific interval operations (transformed to weekly values) and were retrieved from the United States Department of Agriculture Farm Service Agency (USDA-FSA). Disaster declaration data are provided for primary counties and contiguous counties. Only primary counties were used in the analysis. The following types of natural disasters that could potentially disrupt the transportation of crops were considered in the analysis: flash floods, tornados, mudslides, and winter storms. Hurricanes were also initially considered but were dropped from further analysis because they were nonexistent in the sample period and counties. In **Fig 3** the spatial distribution of weekly counts of the examined natural disasters during the study period is shown. Most mudslides occurred in counties across Ohio, while North Dakota counties experienced the highest frequency of tornado occurrences. Flash flooding was notably prevalent in both North Dakota and Ohio counties, while Michigan counties experienced the highest number of winter storms. The disaster data were combined with collection points’ location (i.e., county of operation) to create dummy variables indicating disaster-specific crop transportation disruptions at the county of operation (i.e., *D*). The transportation cost data for corn and soybeans are based on information from publicly available sources for freight rates [35,36], rail rates based on the average on the Minnesota to St. Louis segment [37,38], and St. Louis barge rates [39]. All transportation mode rates are per mile. In each case, data were partial, omitting some weeks or even failing to overlap the full period of our basis data, so rates were related to diesel prices [40] using Ordinary Least Squares (OLS) regressions and these relationships were used to estimate the transportation rates. These data were combined with the least-cost route information from step 2 to calculate crop transportation costs that account for natural disasters. More specifically, using calculated miles and costs for each mode of transportation, a total transportation cost per bushel variable was created as the sum of the products of transportation mode-specific miles and costs (i.e., *C*). The control variables (*X*) include data on historic ethanol intensity measures linked to the corn and soybeans buyers in our dataset, and corn and soybean production. Ethanol intensity is a kernel density function-based estimate that uses the nameplate capacity of ethanol plant located near the sample elevators. Production data are county-specific annual observations that were calculated using acreage and yield data retrieved from USDA Farm Service Agency. Corn and soybean acreage was taken from annual acreage reporting reports [41] and multiplied by the respective commodity yield for the county [42]. FSA yields are calculated using area-based revenue crop insurance products offered through the Risk Management Agency, survey yields reported by the National Agricultural Statistics Service, or the State FSA Committee. **Table 1** presents summary statistics of the variables used in the basis spread estimations.

**Table 1:** Summary statistics of the variables used in the empirical analysis.

	Unit	Soybeans		Corn	
		Mean	St. dev	Mean	St. dev
Basis spread	$/bushel	-1.154	0.373	-0.798	0.25
CostPerBushel	$/bushel	0.807	0.354	0.841	0.352
production	million bushels/county	6.544	6.744	22.377	14.029
ethanol	million gallons/km^2^	0.017	0.014	0.017	0.014
disaster^a^	(0/1)	0.024	0.154	0.011	0.105
flashflood	(0/1)	0.017	0.129	0.009	0.094
tornado	(0/1)	0.004	0.065	0.002	0.044
mudslide	(0/1)	0.002	0.04	0	0
winter storm	(0/1)	0.009	0.095	0.001	0.036
riveradjct-flood^b^	(0/1)	9.55E-05	0.01	0	0
Routechange^c^	(0/1)	0.033	0.179	0.053	0.225

^a^The ‘disaster’ dummy takes the value of 1 if any of the five disasters (i.e., flash flood, tornado, mudslide, winter storm) occurs in the county where an elevator is located for a given week, and 0 otherwise. For the individual disaster dummies, if any of those single disasters occurred in each week, it’s given a 1, else a 0. Mudslides did not affect the sample corn elevators’ routes during the study period (i.e., its mean and standard deviation are both zero).

^b^The ‘riveradjct-flood’ variable takes the value of 1 if an elevator is adjacent to the river and a flash flood occurred in the county that week, and 0 otherwise.

^c^The ‘Routechange’ dummy takes the value of 1 if route changed as a result of a disaster hitting the route, and 0 otherwise.

## 4. Results

**Table 2** and **Table 3** present the results of basis spread estimations. Columns 1 and 2 differ in the level of detail with which natural disasters are incorporated into the estimations. Columns 3 and 4 introduce the interaction between the shipping cost variable and the route change dummy variable. In each case, the Hausman test rejected the random-effects specification, so the results shown in Tables 2 and 3 are based on fixed-effects models.

**Table 2:** Basis spread regression results – Soybeans.

VARIABLES	(1)	(2)	(3)	(4)
CostPerBushel	-0.413***	-0.397***	-0.418***	-0.402***
	(0.0015)	(0.0015)	(0.0015)	(0.0015)
cost_routechange			0.0679***	0.0676***
			(0.0022)	(0.0022)
disaster	-0.320***		-0.321***	
	(0.0029)		(0.0029)	
flashflood		-0.218***		-0.218***
		(0.0035)		(0.0035)
tornado		-0.154***		-0.154***
		(0.0061)		(0.0061)
mudslide		-0.0254***		-0.0250***
		(0.00960		(0.0096)
winterstorm		-0.210***		-0.212***
		(0.0045)		(0.00440)
riveradjct_flood		0.178***		0.176***
		(0.0384)		(0.03830
production	-0.000900***	-0.000836***	-0.000814***	-0.000750***
	(0.0001)	(0.0001)	(0.0001)	(0.0001)
ethanol	8.389***	8.313***	8.067***	7.992***
	(0.1440)	(0.1440)	(0.1440)	(0.1440)
Constant	-0.952***	-0.966***	-0.945***	-0.959***
	(0.0029)	(0.0029)	(0.0029)	(0.0029)
Observations	481,671	481,671	481,671	481,671
R-squared	0.15	0.146	0.152	0.148
Number of elevators	2,304	2,304	2,304	2,304

Standard errors in parentheses.

*** p < 0.01, ** p < 0.05, *  p < 0.1.

**Table 3:** Basis spread regression results – Corn.

VARIABLES	(1)	(2)	(3)	(4)
CostPerBushel	-0.335***	-0.333***	-0.340***	-0.338***
	(0.0014)	(0.0014)	(0.0014)	(0.0014)
cost_routechange			0.0857***	0.0857***
			(0.0025)	(0.0025)
disaster	-0.240***		-0.240***	
	(0.0038)		(0.0037)	
flashflood		-0.268***		-0.268***
		(0.0043)		(0.0042)
tornado		-0.184***		-0.186***
		(0.0085)		(0.0085)
winterstorm		0.152***		0.153***
		(0.0108)		(0.0107)
production	-0.0119***	-0.0120***	-0.0120***	-0.0120***
	(0.0001)	(0.0001)	(0.0001)	(0.0001)
ethanol	4.723***	4.809***	4.357***	4.442***
	(0.138)	(0.1380)	(0.1380)	(0.138)
Constant	-0.329***	-0.330***	-0.322***	-0.323***
	(0.0033)	(0.0033)	(0.0033)	(0.0033)
Observations	274,128	274,128	274,128	274,128
R-squared	0.196	0.196	0.199	0.2
Number of elevators	2,593	2,593	2,593	2,593

Standard errors in parentheses.

*** p < 0.01, ** p < 0.05, *  p < 0.1.

The results indicate that the coefficients of transportation cost per bushel are statistically significant in both the corn and soybean models. Specifically higher cost per bushel leads to a decrease in basis spread. In the soybean models, the basis spread decreases by about $0.40 to $0.42 for each dollar increase in shipping costs per bushel, ceteris paribus, while in the corn models, the decrease ranges from $0.33 to $0.34. These findings align with expectations, as higher shipping costs can reduce local prices relative to a given port price. The direction and magnitude of these effects remain robust across all tested models, as shown in Tables 2 and 3, which vary in the level of detail regarding natural disaster incorporation and include the interaction between shipping costs and route changes, further confirming the reliability of the results.

It is important to note that the cost indicators used are based on general values or broad averages, and may not accurately reflect actual shipping costs. Moreover, even if these indicators closely approximate true prices, an increase in transportation costs might not correspond perfectly to changes in basis. This is because rising transportation costs could have diminishing effects on crop prices at a specific collection point if the crop is rerouted to a different export market, allocated for local use, or placed in storage. In a well-functioning crop distribution system, higher shipping costs in one direction may prompt a search for alternative options.

The coefficients for the interaction term between shipping costs and the route change dummy are estimated to be positive and statistically significant in both the soybean and corn models. When combined with the direct shipping cost results, these findings imply that our average weekly recalculated shipping cost rates tend to overstate the impact on basis spread. For instance, in the soybean model (Table 2, column 3), a $1 increase in rerouting-related shipping costs results in a -0.350 (i.e., -0.418 + 0.068) basis spread change (as opposed to -0.418). One possible explanation is that not all disasters disrupt the transportation network as severely as assumed. Also, the models may overstate the effect of shipping costs on basis spread because they do not account for all potential strategies elevators might use to mitigate transportation cost shocks caused by disasters along the route.

The natural disaster dummy significantly effects both corn and soybean basis spreads. Specifically, basis spread weakens by $0.32 for soybeans and $0.24 for corn when a natural disaster occurs at the collection point. Natural disasters may hamper an elevator’s ability to transport stored crops to ports of exit, resulting in lower local prices relative to port prices. Breaking down the natural disaster dummy into its four components (i.e., flash flood, tornado, mudslide, and winter storm) reveals similar negative effects on basis spreads, with the exception of winter storms in the corn model. Flash floods and winter storms have the largest negative effect on basis spread in the corn and soybean models, respectively. Interestingly, in the corn model, winter storm shows a positive effect on basis spread. These outcomes align with the expected scenarios: a local disaster can impede the movement of crops from the area to NOLA, thereby driving down basis spread. Conversely, such disasters might restrict the influx of crops to the collection point, resulting in a higher basis for the crops that remain available in the affected area. Additionally, the ‘riveradjct-flood’ dummy has a positive effect on basis spread in the soybean models. This may indicate that collection points are unable to transport crops from fields or neighboring collection points to the elevator, leading to higher local prices relative to port prices.

Among the control variables, crop production negatively affects both corn and soybean basis spread. Higher production levels indicate an increased crop supply, which can strain the distribution network and lead to lower local prices compared to port prices. In contrast, the ethanol intensity measure positively influences both corn and soybean basis spreads. Greater local utilization of crops reduces the pressure on the system to transport them to ports for export. Consequently, the increased demand for nearby cash sales of crops can raise local prices relative to port prices. The direction and magnitude of these effects remain consistent across all tested models, which explore varying levels of natural disaster integration and the interaction between shipping costs and route changes, further reinforcing the robustness and reliability of these findings.

For comparison purposes, the models presented in Tables 2 and 3 were also estimated without accounting for unobserved heterogeneity, with the results included in online Appendix S1. While the statistical significance and direction of effects remained consistent for most variables, there were notable exceptions. For instance, the direction of the effects changed for some regressors, including production and mudslides in the soybean models, as well as the winter storm variable in the corn models. Additionally, the coefficients were overestimated in absolute values for most variables in the soybean models, while they were underestimated in the corn models. As expected, these findings indicate that models ignoring unobserved heterogeneity produce inconsistent and biased parameter estimates. For example, the change in the direction of the production coefficient in the soybean models contradicts expectations and economic theory, highlighting the importance of accounting for unobserved heterogeneity to obtain reliable and theoretically sound results.

### 4.1. Basis impacts of disasters at the county level

This section examines the impacts of disasters on the basis spread at the county level, using the coefficients derived from the regressions in Tables 2 and 3. Two distinct effects are estimated: the county of origin effect, which occurs when the disaster directly affects the county where the collection point is situated, and the transportation route effect, which arises when disasters affect counties along the least-cost route from the collection point to the export port. Details on the calculation of these effects are provided in the S2 Appendix in the supporting information.

**Fig 4:**
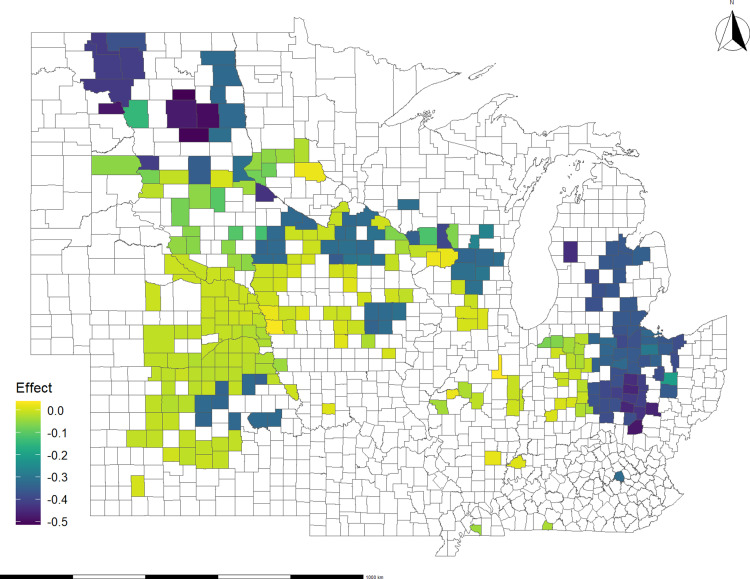
Average impact of all disasters on soybeans basis spread, 2012 – 2020: county of origin and route effect. The effect is in $/bushel.

**Fig 5:**
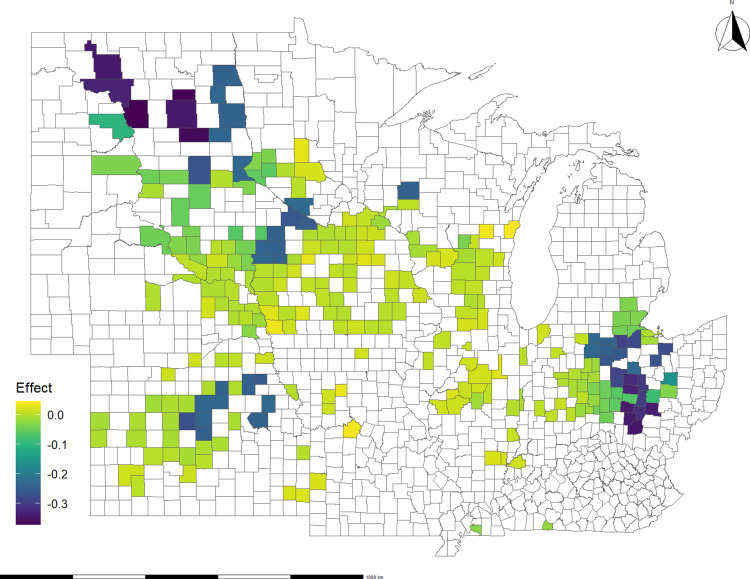
Average impact of all disasters on corn basis spread, 2012 – 2020: county of origin and route effect. The effect is in $/bushel.

A visual representation of the average disaster effects on soybean and corn basis spreads at the county level is shown in Fig 4 and 5, respectively. These maps depict the cumulative effects, capturing the impact at the collection point and along the transportation route. The negative impacts on basis spread run to -$0.50 per bushel for soybeans and -$0.30 per bushel for corn. In the case of soybeans, the counties in Ohio, Michigan, and North Dakota experienced the most negative effects on basis spread during the study period. This aligns with expectations since these states were most heavily impacted by disasters (Fig 3). As for corn, the counties facing the most pronounced negative effects on basis spread include those in Ohio, North Dakota, Kansas, and Iowa. It’s worth noting that these effects exhibit slightly lower magnitude compared to the respective impacts observed in soybeans.

**Fig 6:**
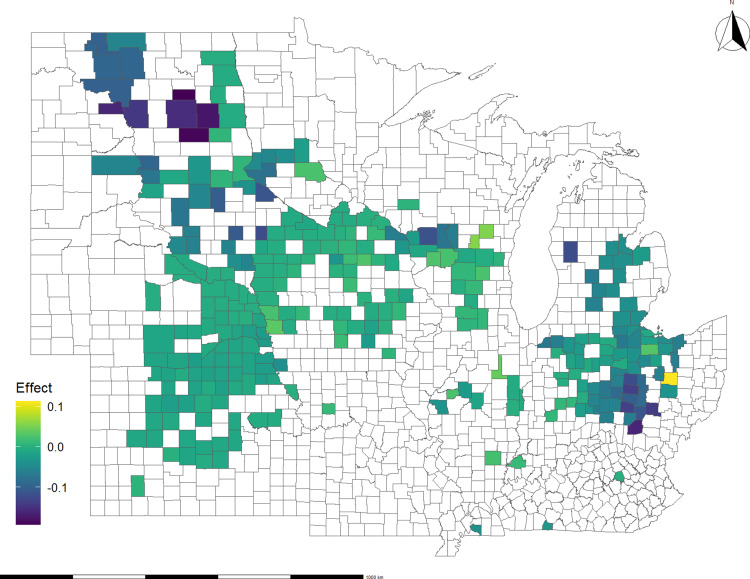
Average impact of all disasters on soybean basis spread, 2012 – 2020: Route effect only. The effect is in $/bushel.

**Fig 7:**
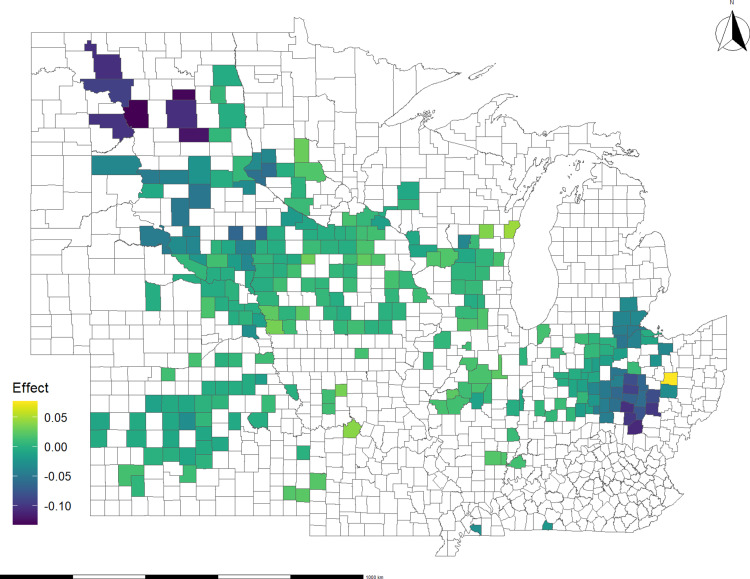
Average impact of all disasters on corn basis spread, 2012 – 2020: Route effect only. The effect is in $/bushel.

Fig 6 and 7 show the along-the-route impact of disasters on basis spread for soybeans and corn, respectively. Counties in Ohio, Minnesota, South Dakota, and North Dakota stand out as some of the most affected areas in both the soybean and corn cases. Additionally, Nebraska, Kansas, and Indiana show several affected counties, though with a lesser degree of impact compared to the states mentioned above. By relating regions where route changes affect basis spread to disaster location data, we infer that disasters, such as flash floods and winter storms, can disrupt crop flows from multiple points of origin. This result highlights the scope and cost of disaster disruptions to the distribution network.

## 5. Conclusions and discussion

This study explores the impact of weather extremes on the basis spread−the margin between buyer and seller prices−for corn and soybean. A three-step procedure is employed to assess the links between weather extremes and basis spread in the United States from 2012 to 2020. First, hypothetical least cost-routes for transporting crops from sample elevators to the port of NOLA are estimated. Second, these routes are re-calculated to account for weather extremes that block crop transportation pathways. Third, crop transportation-related variables and indicators of extreme weather events generated from the previous step, along with control variables, are introduced into an econometric model to evaluate the effect of weather extremes on crop basis spread. By examining how weather extremes can widen the gap between export and farm crop prices, this research highlights potential risks and economic consequences for crop producers and sellers.

The study enhances the understanding of how extreme weather affects market dynamics and price volatility in agricultural commodity markets. Practically, its insights could help the industry make informed location decisions for new facilities, guide Federal and State policy makers in directing transportation funding, and assist transportation planners in mitigating infrastructure vulnerabilities and planning future investments in the crop transportation network.

Results show that weather extremes (modeled using natural disaster indicators) usually have a negative impact on crop basis spread in the U.S. Midwest (measured as local price less port price). Flash floods and winter storms have the most significant negative effects on corn and soybean basis spread, respectively. These disasters had the highest mean frequency of occurrence in the sample, with a combined average of about 2.6% in the soybean model and 1.0% in the corn model (Table 1). This suggests that elevators struggle to move crops to ports of exit during natural disasters in their own counties. Additionally, winter storms positively affect corn basis spread, possibly due to collection points’ inability to bring in crops.

The analysis further reveals that the cost impact of natural disasters along the base least-cost route of sample elevators lowers basis spread. A lower basis spread could harm commodity producers’ income and increase food insecurity by limiting food availability. Therefore, more attention could be focused on mitigating the negative effects of natural disasters on the transportation network that moves crops from the U.S. Midwest to ports of exit. Disasters during the sample period caused shocks of up to -$0.30 per bushel of corn and -$0.50 per bushel of soybeans, with some of this impact linked to disruptions in the crop distribution network. Improvements in transportation infrastructure to enhance resilience against flooding and winter storms might reduce the potential for natural disasters to disrupt this network.

Although this study provides valuable insights into the effect of weather extremes on the U.S. grain distribution network, it is not without limitations. Specifically, the least-cost routes generated are simulated and focus solely on the New Orleans Port. These routes may not fully reflect actual transportation decisions, which are influenced by factors beyond costs, such as market conditions and alternative exit ports. Future research could consider a broader network with multiple transport routes and exit points to better capture the complexities of real-world transportation choices.

Given concerns that extreme weather events will become more frequent, another promising direction for future research could be to estimate the impacts of predicted natural disasters on the U.S. crop transportation network. However, such research is currently hindered by the lack of natural disaster predictions. These predictions would need to be spatially and temporally explicit enough to provide accurate estimates of the climate change costs on the crop transportation network. Second-order approximations could be developed; for example, the average annual impacts of a disaster type (e.g., winter storm, flood) during the historical period could be calculated and paired with general predictions about the future frequency of these events in this region to scale up or down those annual effects. These options fall short in some ways, such as losing location-specific impact estimates, which the current system can provide. Given the impacts of extreme weather likelihood by county and week, this system could estimate collection point basis spread effects.

## Supporting information

S1 Appendix Basis spread OLS regressions that do not account for unobserved heterogeneity. (DOCX)

S2 Appendix Calculating the impact of natural disasters on county-level basis spread. (DOCX)
