# Supplementary material for: Weather extremes and their impact on crop transportation networks: Evidence from U.S. Midwestern elevators
Source: PLoS One. 2025 Mar 31;20(3):e0319815. doi: 10.1371/journal.pone.0319815 (PMC11957334; doi:10.1371/journal.pone.0319815)
Supplement: S1 Appendix — (DOCX) [file pone.0319815.s001.docx]

## **S1 Appendix. Basis spread OLS regressions that do not account for unobserved heterogeneity**

Table S1. OLS basis spread regression - Soybeans

| VARIABLES | (1) | (2) | (3) | (4) |
| --- | --- | --- | --- | --- |
| CostPerBushel | -0.647*** | -0.630*** | -0.655*** | -0.638*** |
|  | (0.00135) | (0.00134) | (0.00136) | (0.00135) |
| cost_routechange |  |  | 0.0972*** | 0.0949*** |
|  |  |  | (0.00241) | (0.00242) |
| disasterdummy | -0.561*** |  | -0.564*** |  |
|  | (0.00307) |  | (0.00307) |  |
| flashflood |  | -0.357*** |  | -0.360*** |
|  |  | (0.00397) |  | (0.00396) |
| tornado |  | -0.445*** |  | -0.447*** |
|  |  | (0.00699) |  | (0.00698) |
| mudslide |  | 0.158*** |  | 0.158*** |
|  |  | (0.0115) |  | (0.0115) |
| winterstorm |  | -0.383*** |  | -0.385*** |
|  |  | (0.00508) |  | (0.00508) |
| riveradjct_flashflood1 |  | 0.0952** |  | 0.0958** |
|  |  | (0.0452) |  | (0.0452) |
| production | 0.00101*** | 0.00109*** | 0.00104*** | 0.00112*** |
|  | (6.77e-05) | (6.80e-05) | (6.76e-05) | (6.79e-05) |
| ethanol | 0.359*** | 0.512*** | 0.315*** | 0.471*** |
|  | (0.0332) | (0.0333) | (0.0332) | (0.0333) |
| Constant | -0.631*** | -0.651*** | -0.627*** | -0.647*** |
|  | (0.00147) | (0.00145) | (0.00147) | (0.00145) |
| Observations | 481,671 | 481,671 | 481,671 | 481,671 |
| R-squared | 0.336 | 0.329 | 0.338 | 0.331 |

Note

Standard errors in parentheses; *** p<0.01, ** p<0.05, * p<0.1

Table S2. OLS basis spread regression - Corn

| VARIABLES | (1) | (2) | (3) | (4) |
| --- | --- | --- | --- | --- |
| CostPerBushel | -0.290*** | -0.290*** | -0.293*** | -0.292*** |
|  | (0.00131) | (0.00131) | (0.00132) | (0.00132) |
| cost_routechange |  |  | 0.0457*** | 0.0456*** |
|  |  |  | (0.00213) | (0.00213) |
| disasterdummy | -0.279*** |  | -0.279*** |  |
|  | (0.00430) |  | (0.00430) |  |
| flashflood |  | -0.207*** |  | -0.207*** |
|  |  | (0.00499) |  | (0.00499) |
| tornado |  | -0.499*** |  | -0.499*** |
|  |  | (0.00986) |  | (0.00986) |
| winterstorm |  | -0.153*** |  | -0.153*** |
|  |  | (0.0127) |  | (0.0127) |
| production | -0.00281*** | -0.00276*** | -0.00285*** | -0.00280*** |
|  | (3.67e-05) | (3.67e-05) | (3.67e-05) | (3.67e-05) |
| ethanol | 2.716*** | 2.669*** | 2.720*** | 2.674*** |
|  | (0.0369) | (0.0369) | (0.0369) | (0.0369) |
| Constant | -0.535*** | -0.536*** | -0.534*** | -0.535*** |
|  | (0.00155) | (0.00154) | (0.00155) | (0.00154) |
| Observations | 274,128 | 274,128 | 274,128 | 274,128 |
| R-squared | 0.173 | 0.175 | 0.174 | 0.176 |

Note

Standard errors in parentheses; *** p<0.01, ** p<0.05, * p<0.1
