# Supplementary material for: Weather extremes and their impact on crop transportation networks: Evidence from U.S. Midwestern elevators
Source: PLoS One. 2025 Mar 31;20(3):e0319815. doi: 10.1371/journal.pone.0319815 (PMC11957334; doi:10.1371/journal.pone.0319815)
Supplement: S2 Appendix — (DOCX) [file pone.0319815.s002.docx]

## **S2 Appendix. Calculating the impact of natural disasters on county-level basis spread**

The basis spread impacts of disasters at the county level include the county of origin effect and the effect along the route. The county of origin effect corresponds to the coefficient of the disaster dummy variable (Column 3 of Tables 2 and 3, third variable). Then, the effect along the route for these elevators was defined as follows:

Effect along the route = [coef_CostPerBushel + (coef_cost_routechange*CostPerBushel)]*add_cost (1)

where, *CostPerBushel* is the shipping cost along the hypothetical least-cost route, accounting for natural disasters; *coef_CostPerBushel* and *coef_cost_routechange* are the coefficients of the cost per bushel variable and the interaction term of cost per bushel and the route change dummy, respectively; and *add_cost* represents the additional expense incurred due to diverting from the least-cost route because of the disruption caused by the disaster along the route. This additional cost is expressed as:

add_cost = c_road*change_miles_road + c_rail*change_miles_rail + c_river*change_miles_rail (2)

The terms *c_road*, *c_rail*, and *c_river* denote the cost per bushel per mile for the road, rail, and river transportation modes, respectively. These costs vary between soybeans and corn. Additionally, the terms *change_miles_road*, *change_miles_rail*, and *change_miles_river* represent the mileage difference between the alternate route (designed to bypass the counties along the route affected by the disaster) and the undisrupted least-cost route for road, rail, and river, respectively.

These effects were calculated separately for corn and soybeans. As the county of origin effect is essentially the estimated coefficient of the disaster dummy, it represents the average effect by definition. The effect along the route is calculated for each impacted elevator and then averaged at the county level (using the elevators operated within each county). The county-specific total transportation disruption effect on basis spread is the sum of the average effect at the county of origin and the average effect along the route.
